# The Implementation of Velocity-Based Training Paradigm for Team Sports: Framework, Technologies, Practical Recommendations and Challenges

**DOI:** 10.3390/sports9040047

**Published:** 2021-03-30

**Authors:** Carlos Balsalobre-Fernández, Lorena Torres-Ronda

**Affiliations:** 1 Applied Biomechanics and Sports Technology Research Group, Autonomous University of Madrid, 28049 Madrid, Spain; 2 Institute for Health & Sport, Victoria University, Melbourne, VIC 3011, Australia; lorenatorres07@yahoo.es; 3 Spanish National Basketball Federation, 28036 Madrid, Spain

**Keywords:** resistance training, monitoring, technology, mean concentric velocity, team sports

## Abstract

While velocity-based training is currently a very popular paradigm to designing and monitoring resistance training programs, its implementation remains a challenge in team sports, where there are still some confusion and misinterpretations of its applications. In addition, in contexts with large squads, it is paramount to understand how to best use movement velocity in different exercises in a useful and time-efficient way. This manuscript aims to provide clarifications on the velocity-based training paradigm, movement velocity tracking technologies, assessment procedures and practical recommendations for its application during resistance training sessions, with the purpose of increasing performance, managing fatigue and preventing injuries. Guidelines to combine velocity metrics with subjective scales to prescribe training loads are presented, as well as methods to estimate 1-Repetition Maximum (1RM) on a daily basis using individual load–velocity profiles. Additionally, monitoring strategies to detect and evaluate changes in performance over time are discussed. Finally, limitations regarding the use of velocity of execution tracking devices and metrics such as “muscle power” are commented upon.

## 1. Introduction

The importance of resistance training (RT) in team sports, either due to the extensive scientific (and empirical) justification, or its impact in improving overall performance [1,2], as well as on minimizing injury risk and in the return-to-play [3,4], is unquestionable. As in other fields, technology can be a source of support, progress and innovation in the RT methodology. However, the advancement in technology for the programming, control and monitoring RT might not always have been accompanied by advances in training methodology, or the best practices of its implementation. The information provided by certain RT tracking systems can have a great impact on the programming and control of training, but especially, on the evaluation and criticism of the training methodology implemented. Thereby, coaches (e.g., strength and conditioning coaches (S&C), physiotherapists) can obtain objective information to analyze whether they are achieving the desired results with their program, how the improvements (or setbacks) occur, and thus, can improve their general knowledge of the strength training paradigm.

Two of the most frequent indicators used as a reference to prescribe RT have been the 1-Repetition Maximum (1RM) and the maximum number of repetitions at a given percentage of this weight (n1RM%) [5,6]. However, despite being useful to some extent for an apparent individualization of training, these procedures present limitations that made the scientific and applied community explore other strategies, leading to the design of strength training programs. A still emerging method, although not *new*, is the velocity-based training (VBT), representing both a less invasive and a more optimal method to prescribe RT based on velocity of execution. This is a key element in most sport disciplines, either by the requirements of the sport (e.g., sprinting, accelerating/decelerating, jumping, throwing, kicking) or as an indicator of intensity (e.g., neuromuscular demands) [7,8]. There are reports of German weightlifters using linear transducers in the 1970s [9]. Since that time, research on the benefits of lifting velocity to prescribe and monitor RT has been conducted [8,10,11,12]. Being able to objectively monitor RT sessions, adapt the goals individually, or provide feedback in real time on a daily basis is paramount, especially when working with elite players. In addition, compiling information will allow a critical analysis based on objective data on the implemented methodology, and thus enable the necessary adjustments and advancements in the knowledge of the strength training theory. However, there are still some confusion, misinterpretations and wrong practices when implementing VBT in team sports. Therefore, the aim of this article is to provide a comprehensive overview of the main benefits, technologies and best practices when implementing VBT programs in team sports.

## 2. The Challenges and Drawbacks of the 1-Repetition Maximum Paradigm

The value of 1RM represents the maximal load (mass) that the athlete can lift, in a concentric dynamic action, with proper form, once but not twice [13]. Thus, performing a 1RM is considered a maximal dynamic effort. Once this value is known, programming of RT is done prescribing relative loads to the individual’s 1RM [14,15]. However, this methodology comes with certain disadvantages.

It is well known in the powerlifting community that experienced lifters are able to move their 1RM load (mass) at a lower speed than their less trained or experienced counterparts, especially in complex multi-joint exercises. Either due to inexperience, not making a truly maximum effort or having experience but avoiding situations of risk of injury using maximum loads, the 1RM is often poorly measured, which implies that the consequent prescribed load (percentage-based training; *n*RM%), or velocities for each 1RM%, will not be adequate.

Moreover, as happens with other measures of physical performance, the 1RM could vary on a daily basis due to several uncontrolled factors (e.g., fatigue, mental preparation, sleep, stress or nutrition) [16]. Hence, in order to prescribe a precise load for each session, the 1RM should be tested at the beginning of every RT session to adjust loads to the actual (current) daily maximal capabilities of the athlete. However, since conducting an actual 1RM test before each training session is utopic, traditional percentage-based approaches have been used, where the 1RM is assessed at the beginning of a training cycle, the %RM is calculated and the RT program periodized for the subsequent weeks according to it [12,17]. The main drawback with this approach is that, since the actual 1RM could vary from one session to another, the subsequent prescribed loads (mass) for the cycle would likely represent a different effort to the initial loads (%RM) programed based on the original 1RM. As an illustration of this potential discrepancy, Table 1 displays a RT load progression over a period of 8-weeks, with one example based on the 1RM assessed prior to the training cycle, and an example based on daily estimations using load–velocity profiles.

Additionally, the effort that represents each 1RM percentage differs between exercises [18,19], where, for example, the 85% 1RM represents a different effort in bench press vs. back squat. Furthermore, the load that represents a %1RM still requires performing repetitions to failure, and extensive research has shown that performing repetitions to muscular failure can impair performance, at specific times of a periodization program, due to an excessive production of fatigue, thus making it inappropriate for team sports training [20].

## 3. Technologies to Track Velocity for Resistance Training

Due to the growing interest in VBT, there has been a proliferation of devices measuring velocity for RT, from motion capture systems (MoCap), linear transducers and accelerometers, to low-cost smartphone apps. In addition, this phenomenon has been accompanied by a number of studies conducted on the validity and reliability of some of these novel technologies to measure velocity [21,22,23,24,25,26,27,28,29]. However, each of these technologies has advantages and disadvantages, and practitioners tasked with managing the systems have the responsibility to understand the pros and cons of the different systems, as well as using a systematic process in the data collection, being knowledgeable on best practices regarding implementing VBT, and applying critical thinking when assessing their RT methodologies.

Team sports rarely require static, linear or single-joint movements. Rather, it is the opposite in that there are not only complex movements but also ones performed in highly unpredictable environments and with opponents (involving contact, perturbations, collisions, and such). Velocity tracking systems are still far from being able to measure with ecological validity; most are designed to measure *traditional* strength exercises, connected to equipment, or to certain body segments.

**Figure 1:**
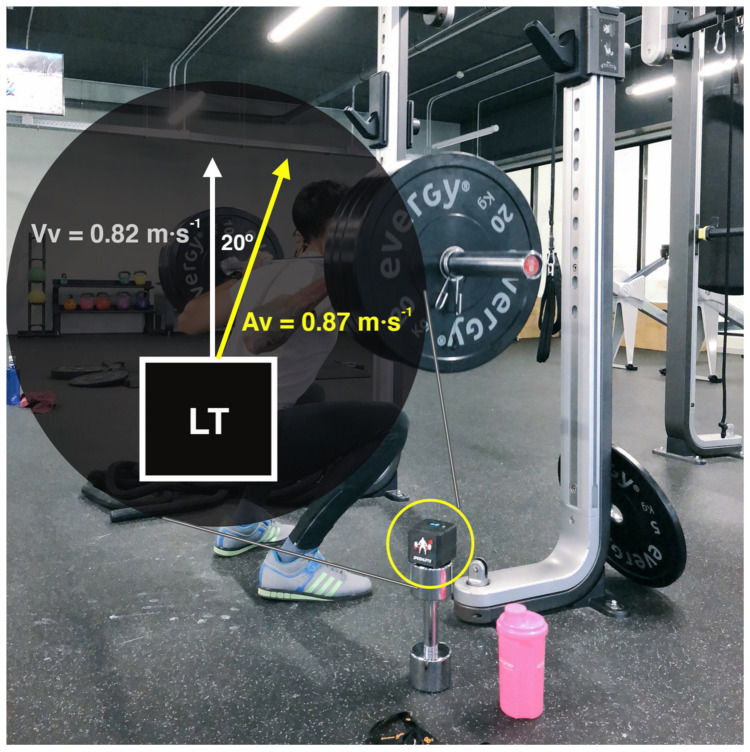
Generic linear transducer showing a deviation of 20° from the vertical during a lift. For a registered mean velocity of 0.82 m·s^−1^, the actual magnitude of the resultant mean velocity vector would be 0.87 m/s, as calculated using simple trigonometry (Actual velocity = Registered velocity/cos (angle). Most of the load–velocity relationships analyzed in the scientific literature are conducted with exercises performed in Smith machines, since this equipment guarantees a complete vertical motion of the barbell. This, however, reduces the ecological validity of the load–velocity relationship itself, since these profiles can differ if the lift is performed with free weights (where horizontal displacements occur) or with a Smith machine.

Linear transducers are probably one of the most popular technologies used in the implementation of VBT to date. One of the downsides is that they are limited to measure vertical motion only [30,31]. Most commercial models available in the market just measure the vertical component of the velocity vector, meaning that any horizontal or lateral motion during the lift would be (and should be) omitted (Figure 1). Horizontal displacements do occur when using free weights even in “vertical” exercises like the back squat [32,33]; hence, a proper exercise form and technique should be guaranteed, and exercises with a horizontal or angular momentum component avoided.

In most cases, linear transducers are attached to one end of a barbell, and thus they actually register the velocity of that part of the bar, which could differ from the velocity of the other end if the athlete did not lift it *perfectly*, parallel to the ground. Lifting an unbalanced barbell does happen in practice, and S&C coaches using linear transducers should be aware of this fact. To reduce this type of error in the measurement, some studies have used two linear transducers, and reported the mean velocity registered from the two devices [30,32]. This may not be feasible in applied contexts; an alternative support may be having a visual control of the form of execution (objective or subjective) and exclude incorrect repetitions. It is then recommended that the device is placed consistently on the same side.

Trying to solve some of the aforementioned limitations, different novel technologies such as accelerometers or smartphone apps have emerged as a cost-effective and practical alternative to measure movement velocity in resistance exercises, and studies have shown that they can do it in a valid and reliable way [22,25,28,34]. For example, certain accelerometers can track velocity in exercises with horizontal displacement, while apps have shown reliable measurement of mean concentric velocity in cable-based exercises by video-recording the vertical ascent of the machine’s weight stack [35]. However, these devices come with their own limitations. While they have shown moderate to good validity and reliability, accelerometers had the lowest accuracy when compared with other devices, such as linear transducers, apps, MoCap or gold-standard instruments [22,28]. A popular app [25] has presented validity, reliability and accuracy higher than accelerometers; however, since it is video-based (frame-to-frame slow motion navigation), the measurements are not provided in real time. When comparing different linear transducers, studies have observed differences in absolute velocity outputs [28]. Thus, when interpreting VBT research (especially when looking to raw velocity data) practitioners should interpret results carefully; it is advised to not use devices from different manufactures interchangeably.

Another common misappropriation of technologies to track movement velocity is its application for the measurement of power or “muscle power”. Power is important in sport because it is an expression of performance, the consequence of the force applied in a given time in a given action, which is paramount in many sport activities [36,37,38]. It is a product of force and velocity, but depending on how force and velocity are calculated, the resultant “power output” can vary [30,36]. How force is calculated has a great impact on the “power output”, producing contradictory findings that can confuse S&C coaches. For example, studies that have analyzed the load that maximizes power output in different exercises have found heterogenous results, with wide observed ranges (from 30 to 80% 1RM) that still make it challenging to come to a consensus about which load elicits the maximal power output [30,39,40,41,42]. Likely, the main reason for those discrepancies is the method used to calculate the force. The gold standard to measure force production in dynamic actions, such as jumping or lifting, are force plates [30,43]; they are designed to continuously measure force exerted on them, and calculate the force applied to the *system mass* (e.g., the bodyweight of the athlete plus the mass of the barbell), when performing the exercise. Most velocity tracking systems (e.g., linear transducers, accelerometers, MoCap) do not take into account the athlete’s bodyweight (mass) in their “power” calculations; therefore, reviewing whether they are included in the force calculation is advised). Instead, they calculate it by the differentiation in the velocity measured to obtain acceleration, and afterward multiply it to the mass of the barbell [41,43]. This method has been proven to underestimate the actual power (force applied at a given velocity), when pushing to the ground when jumping [44]. Studies using “barbell power” measurement have observed that the load that maximizes power in vertical jumps, or ballistic bench press throws, are much higher than those using the “system power” [36,41,45,46]. For example, if the body mass (aka system mass) is considered in the calculation of force, the load that maximizes power output is close to 0 kg (i.e., unloaded jump), while, when considering only the barbell mass, the load that maximizes power output is close to 100% of body weight for the external load [46]. Thus, the practical recommendation to maximize “power output” during vertical jumping can be completely different if the system mass or the barbell mass is used in the calculation of force. Although “barbell power output” could be of great interest for athletes whose main activity is to apply maximum power to an implement (e.g., throwing), it is our opinion that it is not the most appropriate method when assessing power production capabilities in team sport players. Nonetheless, if the goal the S&C coach is to measure “power output”, they are encouraged to use force platforms and include the total mass (athlete body mass and external mass) in the computations from the linear transducer.

Finally, it is worth noting that different manufacturers might report different velocity metrics, with the most popular being MVC, mean concentric velocity of the propulsive phase (MPV) and/or peak velocity (PV) [8,29,47]. While it has been proposed that MPV can better represent the velocity production ability of the athlete [48], it has also been observed that mean concentric velocity (MCV)is as reliable as MPV [47], and, from a technological point of view, simpler to measure. It is also worth mentioning that, considering that MCV, MPV and PV are different (generally, MCV < MPV < PV), these metrics should not be used interchangeably. For standardization purposes, in this document we refer to MCV when citing velocity of execution.

## 4. Determination of Individual Load–Velocity Profiles in Team Sport

Early technology used to measure velocity was expensive, not accessible to the vast majority of practitioners and mainly constrained to measure vertical motions (e.g., linear transducers). Nowadays, there are more accessible devices, capable of measuring the velocity in different types of equipment, which allow the assessment of numerous exercises simultaneously (e.g., a player being able to track a session in multiple exercises or players working at the same time in different stations). The latest point, working with large squads, is probably one of the biggest challenges when it comes to measuring (or *testing*) in team sports, since time can be a constraint. Velocity tracking systems are evolving, making assessment procedures less time consuming for both coaches and players. However, there are some key aspects to take into account to successfully implement VBT during the RT session with team sport athletes.

The vast majority of the exercises analyzed in the VBT literature are barbell-based [8,49,50,51], and researchers have found an almost a perfect association between mean concentric velocity (MCV) and load (%1RM) in several exercises, such as bench-press, back squat, deadlift or hip-thrust. Some studies have also observed that the load–velocity relationship is well-fitted in machine and cable-based exercises (e.g., the leg extension) [35]. What is important to highlight here is that the load–velocity relationship is exercise-dependent [18,52,53], since the load that represents a certain absolute velocity can vary greatly between exercises (Table 2). Thus, the use of absolute “velocity zones” is discouraged, since a certain velocity (e.g., 1.0 m·s^−1^) can represent a low load (mass) (e.g., when performing a back squat) or a near maximal load (e.g., when performing an Olympic Snatch). Including a battery of one lower-body, one upper-body, and/or full body multi-joint barbell exercises in the assessment is recommended.

The load–velocity profile is created by calculating a regression that fits the velocity (data points) with different loads (absolute or relative), where the more loads assessed, the higher the accuracy of the profile. However, including a high number of loads (e.g., 10 loads) would be time consuming and impractical when testing a large squad of players. Moreover, in order to avoid fatigue and guarantee that the athletes are performing the lift at their maximal velocity capabilities, a passive rest of 3 to 5 min between loads is needed. Consequently, it is recommended to select a number of loads (mass; data points) that is time efficient but, at the same time, that guarantees a regression fit. It was observed that profiles created from two loads can be as reliable as those created from six loads, as long as the loads used are properly selected; in this case, it seems that a light load (about 40% 1RM) and a heavy load (about 80% 1RM) are the best options [11,54]. However, if one of the two loads are not properly performed (i.e., athletes not applying their maximal intended velocity), the results can be drastically altered. Therefore, we recommend performing between 4 and 6 sets with incremental loads to guarantee that the results are reliable (Table 3).

Research has shown that subjective scales are highly related with MCV in different resistance exercises [55,56,57]. For example, it has been observed that both the Repetitions in Reserve (RIR) (calculated as the number or % of repetitions performed with respect to the maximum number of possible repetitions) and the Rating of Perceived Exertion (RPE) scores are associated with velocity loss with different loads [58]. Hence, accounting for the RIR and/or RPE can be useful tools when programming, and for understanding the mechanical fatigue that the athlete is experiencing without having to measure the velocity drop over the set. Moreover, it has been shown that the inclusion of RIR with the movement velocity in a multiple regression improves the accuracy of the load–velocity profile [59]. While practitioners should ideally measure MCV in most of the main exercises, prescribing RT using RIR can be a suitable solution in those exercises where calculating the load–velocity profiles might be of less interest or not suitable, such as dumbbell, cable, kettlebell exercises, and the like. Moreover, it has been observed in youth basketball players that training using a RIR approach is more suitable than conducting traditional repetitions to failure when velocity is not used to monitor training load [60]. Table 4 presents a VBT training prescription where some exercises are prescribed using velocity, and others are based on RIR.

## 5. Considerations for a Successful Implementation of a Velocity-Based Program

The velocity of execution (during the concentric phase of dynamic exercises) is a key training variable in sports performance. In the following, we present some important remarks when implementing VBT:

- The most relevant consideration when training based on the velocity of execution (VBT) is that velocity must be the maximal intended. If the athlete does not perform with a maximal intended velocity (regardless of the load (mass) or %1RM), the results would be underestimated [61].
- VBT is not specific for velocity-oriented sessions or exercises; it is a RT methodology based on the velocity of execution, used to prescribe, monitor and analyze RT. The velocity of execution can be used at different %RM (percentage-based training), including heavy loads; choosing appropriate and key exercises is paramount.
- While the 1RM might vary within days, the velocity at each 1RM percentage when individual load–velocity profiles are computed is very stable [62]. Therefore, the velocity of execution with a fixed absolute load can be a good indicator of effort and actual (current) performance.
- The velocity measured is both load (%RM) and exercise dependent. It has been shown that different exercises have unique velocities associated with each percentage of the 1RM (Table 2).
- Individual load–velocity profiles can differ between genders, age or training status [49,63], and they have shown higher reliability than generalized profiles created from normative data [64,65].
- Practitioners can benefit from combining objective and subjective scales to improve the accuracy of the training load prescription. As observed in a recent review on the topic, both objective (i.e., mean concentric velocity) and subjective measurements (i.e., repetitions in reserve) can help to enhance muscular strength by prescribing training loads that take into account the athlete’s daily fluctuations in performance or fatigue [66]. Moreover, it has been observed that the combination of MCV and RIR increases the accuracy of the 1RM estimation in comparison with using MCV alone [59].
- The mean velocity might represent different levels of effort depending on the anthropometric profile. It is paramount to create individual load–velocity profiles for each athlete, especially if the players within a team have large differences in their anthropometrics. This can be especially relevant in sports like basketball, where limb lengths can have a wide range between players. For example, two players can lift the same *relative* load (i.e., %1RM) in the bench press at 0.8 m·s^−1^, but player-A barbell’s displacement is 0.35 m, while for player-B it is 0.6 m; this means that player-B has produced the same velocity as player-A, but in almost twice the time (0.75 s for player-B vs. 0.43 s for player-A). Thus, player-B spends more time under tension, meaning that the overall effort for the same velocity might be higher for player-B than for player-A.

## 6. Prescribing and Monitoring Training Loads with VBT

**Figure 2:**
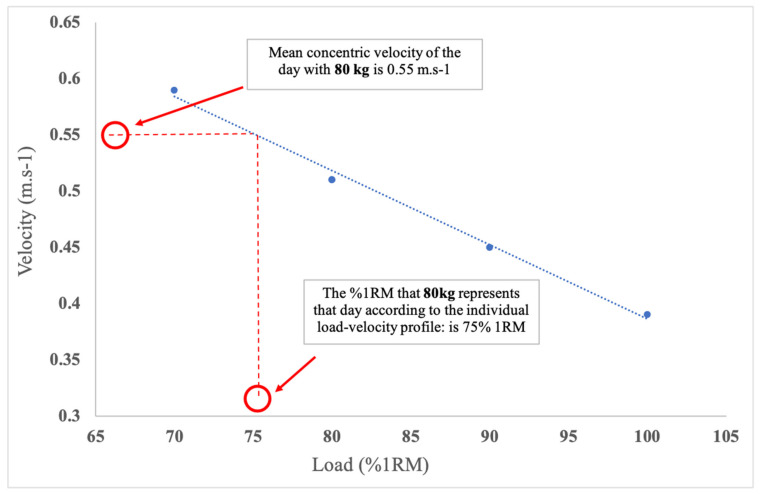
Estimation of daily 1RM scores on the back squat using individual load–velocity profile of the player. In this example, if the athlete has lifted the 90 kg at 0.49 m·s^−1^; according to his individual profile, that represents his 85% 1RM. Consequently, the theoretical 1RM of that day would be 105.6 kg.

There are certain methodological aspects when using velocity of execution during RT that can be relevant as indicators of effort and for monitoring training on a daily basis. As a starting point, practitioners could choose key exercises for the training cycle, and determine a load–velocity profile for those. Secondly, the relative intensities of the training cycle (e.g., %RM) should be defined. Thereafter, measure the velocity in the first set of an exercises to establish subsequent loads for the day; to do that on a daily basis, practitioners would choose a fixed load (mass) for the warm-up, perform one set of 1–2 repetitions and measure the MCV. Once the velocity at the fixed load is known for that day/exercise, adjust loads to the actual (current) daily maximal capabilities of the athlete that represents the intended velocity for the programmed intensity (%RM). Figure 2 displays an example of a player’s load–velocity profile, the speed for the warm-up repetition, and the corresponding %RM. The faster the velocity, the lower the percentage of the 1RM that the load represents.

Finally, prescribe the number of repetitions per set by either: (i) the velocity loss thresholds within the series, (ii) the repetitions in reserve, or (iii) using subjective scales. A relevant use of VBT when prescribing RT sessions is being able to identify neuromuscular fatigue and modify the number repetitions the athlete should perform during the set [10,67]. It has been observed that velocity loss during the set is highly related with markers of fatigue such as lactate or ammonia [68]. Studies have observed that low velocity loss thresholds (i.e., 10–20% loss from the fastest repetition) can produce similar improvements in physical performance with significantly lower training volume than higher thresholds (i.e., 40% loss or more) [67,69] On the contrary, if the RT goal is to maximize muscle mass, research has shown that lifting close to muscular failure is more adequate [70,71], and it has been observed that high velocity loss thresholds (i.e., 40% or more, which means going closer to failure) would be more appropriate to increase hypertrophy [69].

The goal of strength training in sport is to improve force, velocity and power output production capabilities [37]. A positive training program would change MCV with a fixed absolute load/s or the load that can be lifted at a fixed speed. It has been shown that load–velocity profiles (i.e., the velocity associated with each %1RM) can be altered after a period of training of 4 to 6 weeks [49,72]. Thus, it is recommended to re-assess the load–velocity profile every ≈4–6 weeks, or after specific cycles of RT, in order to evaluate the effects of the RT. However, the training capacity of the athlete might vary on a daily basis, and that is why the measurement of the velocity execution in the first series or repetitions of an exercise is useful for the programming of each training session. Some velocity tracking systems (e.g., My Lift App, Madrid, Spain) include calculations within their algorithms that estimate 1RM on a daily basis by measuring individual load–velocity profiles, as explained in Figure 2.

**Figure 3:**
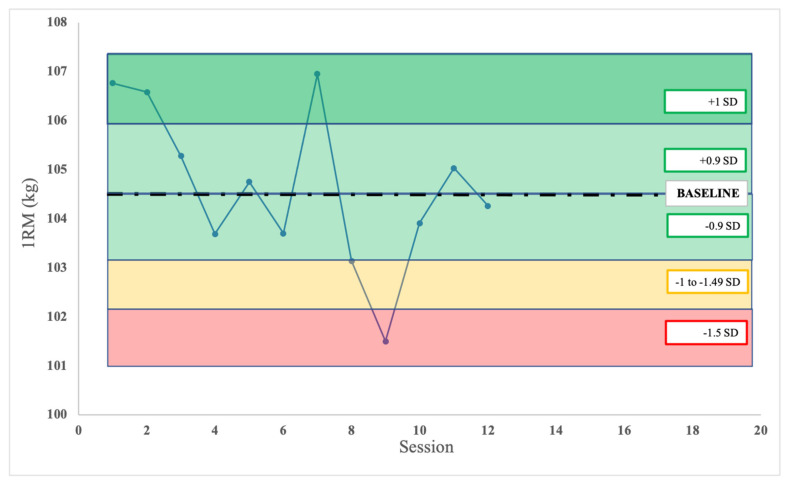
Variation of bench-press 1RM over a period of 8 weeks. The black dashed line represents the baseline score (calculated as the average score of the previous 2 months), while the dark green, green, yellow and red shadowed areas represent +1 standard deviations (SD), +0.9 to −0.9 SD, −1 SD and −1.5 SD with respect to the baseline, respectively. Note that when there is no data in the weeks before the start of the training program, this approach cannot be used until enough data is collected.

Finally, in order to analyze the evolution of the players, we propose to “*put the I back in team*”, as nicely discussed in Ward et al. [73]. A current limitation in the strength and RT research is that most results provide group averages. Yet, to better optimize players’ performance, an individual analysis is paramount. In order to analyze individual trends, proper statistical techniques should be implemented. Ward et al., in their 2018 study, proposed different statistical strategies to analyze the evolution over time of a single athlete. Figure 3 provides an example of day-to-day variation in bench-press performance over 8-weeks.

## 7. Conclusions

This manuscript aimed to provide a guideline to better implement VBT in team sport settings. The ultimate goal of RT in team sports is to improve the force production applied at a certain load (mass), at a given velocity and/or during a certain time. The velocity of execution is a reliable indicator of effort for programming and monitoring training, as well as for managing fatigue, both on a daily basis and in long term periodization.

The advance in tracking systems allows measurement of an athlete’s velocity of execution during RT sessions, in different exercises during the same session, for a more comprehensive monitoring of the program. However, managing large squads during every training session can be challenging; how to best use technology in an efficient way, and educate coaches and players in its use, would be paramount.

One consideration when using VBT tracking systems is the lack of research in their use in different strength training modalities, such as eccentric-overload training, pneumatic resistance or iso-inertial devices, and elastic-based equipment, where a velocity–effort (e.g., load (mass), %RM) relationship might not be the primary RT training goal with these tools.

Programming using VBT does not automatically imply programming effectively. The prescription, control, monitoring and evaluation of a RT using VBT requires a clear understanding of certain physics concepts, as well as strength training methodology. The VBT implementation should not be seen as an accessory to traditional RT or as a purely motivational tool for providing real-time feedback (the latter, without a doubt, being a great tool). The use of velocity in RT is a training paradigm, where specific strength goals and adaptations can be achieved by properly programming and tracking velocity of execution. It also allows for an objective quantification of the resistance training loads. Implementing VBT to monitor players’ performance can support decision-making processes, particularly the making of informed decisions when programing RT, and improvements in knowledge of the strength training paradigm.

## Figures and Tables

**Table 1:** Comparison of the load associated with 70% 1-Repetition Maximum (1RM), using the pre-test value (session 1) or actual daily values.

	Session 1	Session 2	Session 3	Session 4	Session 5	Session 6	Session 7	Session 8
**1RM (kg)**	130	---	---	---	---	---	---	---
Load @ 70%1RM (kg)	91	91	91	91	91	91	91	91
**Actual daily 1RM**	130	132.5	130	135	137.5	132.5	135	140
Load @ 70% 1RM (kg)	91	92.75	91	94.5	96.25	92.75	94.5	98

In the pretest session (i.e., Session 0), the 1RM of the athlete represented in this table was 130 kg in the bench-press exercise. Those 130 kg were used as a reference in the “pretest programming”, while daily 1RM scores estimated by measuring barbell velocity were used in the “daily programming”. Note that in the pre-test programming, every session would have been performed with 91 kg (i.e., the 70% of the pre-test 1RM), but if daily variations would have been taken into account, the actual load would have variated on a daily basis.

**Table 2:** Velocities for different %1RM from an individual load–velocity profile of one player, for bench-press, back squat, deadlift and pull-up exercises [8,18,52,53].

Load (% 1RM)	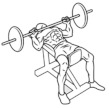	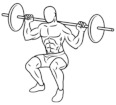	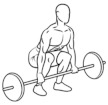	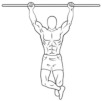
40%	1.03	1.21	1	0.93
45%	0.96	1.14	0.94	0.88
50%	0.89	1.06	0.87	0.83
55%	0.82	0.99	0.81	0.79
60%	0.75	0.91	0.75	0.75
65 %	0.66	0.84	0.69	0.7
70%	0.60	0.77	0.62	0.65
75%	0.53	0.69	0.56	0.60
80%	0.46	0.62	0.5	0.56
85%	0.38	0.54	0.44	0.51
90%	0.31	0.47	0.37	0.47
95%	0.24	0.39	0.31	0.43
**100%**	**0.17**	**0.32**	**0.25**	**0.38**

**Table 3:** Protocol to determine the load–velocity profile; bench press exercises as an example.

1	**Loads selection:** Select 4–6 incremental loads, using velocity as a reference, starting approximately @ 30–40% 1RM (>1.15–1.20 m·s^−1^) and until approximately @ 75–80% 1RM (≈0.55–0.45 m·s^−1^), or higher, if needed.
2	**Set (0):** Warm-up @ 20–30% RM. If %RM is unknown, use an easy load or a load that can be lifted approximately @ >1.20–1.25 m·s^−1^. This set can be used to adjust the 1st load for the incremental test.
3	**Set 1–4/6:** Athlete performs 2 reps with each load (1 rep when using *heavier* loads is allowed), pushing the barbell **as fast as possible**. The fastest repetition is the one used for the calculations. When measuring only the concentric phase, a brief 1–2 s barbell stop on the chest is needed. Rest ≈2–3 min between sets, ideally passively. Other interspersed exercises can be performed, but these should not involve upper body or fatigue.
4	**Load increment between sets:** Ideally, the load should be increased similarly (for example, by adding 5 or 10 kg in each new set, or a similar velocity lose), or a proportional decrease in velocity (≈0.10 m·s^−1^).
5	**Assessing progression:** After a training cycle, velocity is re-measured with the absolute loads used in the first incremental test. As an alternative, practitioners could: (i) select an absolute load (mass) and assess the change in velocity for that particular load, and/or (ii) measure at a certain velocity, and see with which load is used compared to the start of the cycle.
**Notes**	
It is not recommended to perform a load that implies >90–95% 1RM. For example, in the bench press exercise, research has shown that the velocity of 90–95% 1RM is ≈0.35–0.40 m·s^−1^). If in one particular set the drop in velocity is significantly higher than with the previous load, increase the load to a lesser extent. After the assessment is completed, calculate the coefficient of determination of the load–velocity profile. If R^2^ is lower than 0.92, in our experience it is recommended to review the data to find and repeat the load/s that was/were not properly performed. Typically, a correct test always has a coefficient of determination >0.96.	

**Table 4:** Example of a distribution of load (mass) and the number of repetitions per set using a velocity-based approach.

Exercise	Load (kg)	Sets	Repetitions
Bench-press	80 ^#^	3	6 (Until a 20% of velocity loss was achieved)
Back squat	100 ^#^	3	5 (Until a 20% of velocity loss was achieved)
Pendlay row *	80	3	6 (RIR 2)
Hip thrust *	120	3	5 (RIR 3)
Shoulder press *	50	3	6 (RIR 2)
Leg press *	160	3	5 (RIR4)

Notes: * Exercises where no load–velocity profiles are calculated; the absolute load is prescribed as the load that makes it possible to reach the prescribed RIR (i.e., Repetitions in reserve). ^#^ The load is calculated using individual load–velocity profiles.

## Data Availability

Not applicable.
